# Mechanotransduction by nuclear envelope tension

**DOI:** 10.1080/19491034.2025.2600901

**Published:** 2025-12-16

**Authors:** Sriivatsan G. Rajan, Pere Roca-Cusachs, Philipp Niethammer

**Affiliations:** a Cell Biology Program, Memorial Sloan Kettering Cancer Center, New York, NY, USA; b Institute for Bioengineering of Catalonia (IBEC), The Barcelona Institute of Science and Technology (BIST), Barcelona, Spain; c Department of Biomedical Sciences, University of Barcelona, Barcelona, Spain

**Keywords:** Cytosolic phospholipase A2, mechanobiology, membrane, nuclear deformation, nuclear envelope, nuclear pore complex, nuclear transport, nucleus, tension, YAP

## Abstract

Mechanotransduction mediated by the tension in lipid membranes is a well-established paradigm. This has been studied largely in the context of the plasma membrane, but recent work shows that it applies also to endomembranes, and specifically to the nuclear envelope. Here, we review membrane tension-mediated mechanotransduction at the nuclear envelope by focusing on its two best characterized modes of action: the cytosolic phospholipase A2 (cPLA_2_) pathway, and nuclear pore dilation. We discuss the mechanisms involved and their physiological implications. Finally, we discuss how nuclear envelope tension can be controlled and measured, and how its properties enable mechanosensing with different context-dependency than that of the plasma membrane. These properties apply to cPLA_2_ and nuclear pore complexes but potentially also to many other mechanosensors yet to be discovered.

## Introduction

Lipid membranes play an essential role in cell biology, in terms of both chemistry and mechanics. Mechanically, forces applied through a membrane (either from the extracellular space or in between compartments separated by endomembranes) will deform the membrane, which can then alter the mechanical tension transmitted through it (membrane tension). Accordingly, molecular mechanisms able to detect membrane tension are well placed to act as mechanosensors, i.e., switches converting mechanical cues into downstream biochemical responses. This paradigm is well established for the plasma membrane (PM), particularly for regulating ion flux through mechanosensitive ion channels such as Piezo1, TREK, and TRAAK [1]. By contrast, the view that the nuclear envelope (NE) senses the physical environment is still emerging.

The PM and NE are the two largest sensory surfaces of a cell. Both detect mechanical deformation, but they operate in different regimes. By being in direct contact with the extracellular space, the PM can sample small, local deformations across the entire cell surface. In contrast, the NE – reinforced by a shock-absorbing lamina and separated from the cell surface by the cytoplasm and cytoskeleton – is better suited to sensing large deformations of the cell body. The integration of these overlapping yet non-redundant inputs enables cells to distinguish peripheral from central, and harmless from potentially life-threatening, mechanical stimuli, thereby guiding migration, proliferation, and differentiation.

Here we provide a timely update on two mechanotransduction mechanisms that are relevant to the nucleus: (i) the nuclear cPLA_2_ pathway and (ii) the nuclear pore complex (NPC). We also briefly overview the regulation of and experimental measurement methods for their shared key trigger: (iii) nuclear-envelope tension. Interestingly and analogously to the case of the PM, a key aspect discussed in here is whether NE tension matters at the level of the lipid bilayer, the underlying lamina, or both. For broader treatments of cellular and nuclear mechanotransduction, we refer readers to earlier comprehensive reviews [2,3] and to the other articles in this Nucleus special issue.

### The nuclear cPLA_2_ pathway

Mechanotransduction at stretched membranes is often treated as synonymous with mechanosensitive ion channel activation, yet many other mechanisms exist, mediated by membrane unfolding, membrane tension, membrane curvature, or membrane domain rearrangement [4]. In particular, reconstituted systems showed decades ago that peripheral membrane enzymes also respond to bilayer tension [5–9]. The physiological relevance of this concept became first evident in zebrafish wound-healing experiments, which identified cytosolic phospholipase A_2_ (cPLA_2_; PLA2G4A) as a key mechanosensor at the inner nuclear membrane (INM) [10,11].

Zebrafish skin is bathed in low-osmolarity water; in humans, desalination in salivary ducts creates a comparable hypotonic milieu in the upper digestive tract, with up to 2 l of hypotonic saliva [12] per day. Zebrafish tail fin injury under hypotonic bathing conditions induces local osmotic shock and cell swelling accompanied by the recruitment of cPla_2_ to the INM [11,13]. Preventing shock in zebrafish by immersion in isotonic solution abolishes rapid antimicrobial leukocyte recruitment impairing post-infection survival [10,11,14]. These observations established cPLA_2_-dependent nuclear membrane mechanotransduction (NMMT) as a wound-detection mechanism that converts osmotic swelling into inflammation.

**Figure 1.**
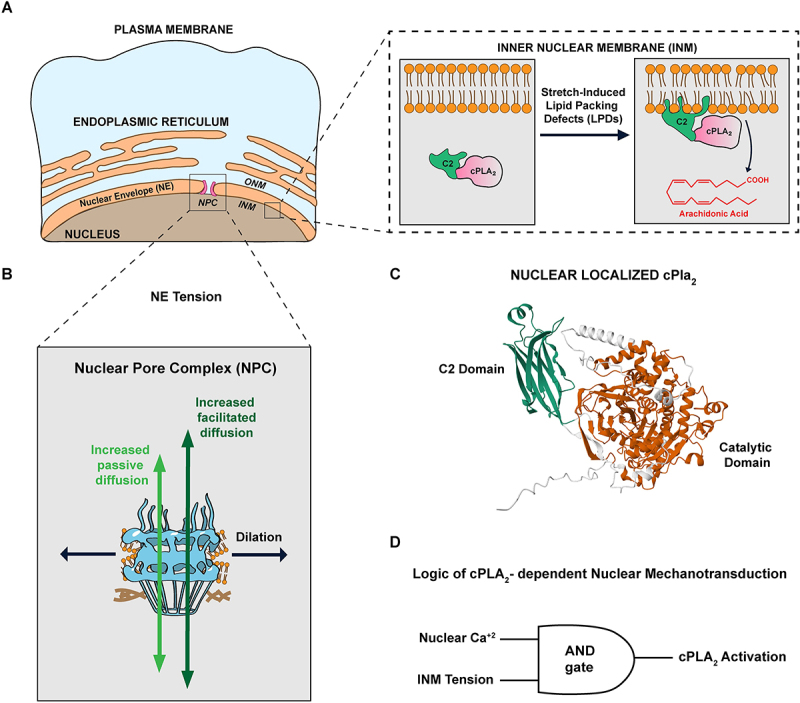
Activation of cPLA_2_-dependent and NPC-dependent nuclear mechanotransduction (A) Schematic shows how mechanical stretching induces the adsorption of cPLA_2_ into the hydrophobic sites of the inner nuclear membrane (INM) followed by arachidonic acid release. (B) Tension applied to the NE dilates NPCs, increasing passive and facilitated diffusion to different extents. This alters the transport properties of shuttling molecules such as transcription factors. (C) Structure of zebrafish cPla_2_ predicted by Alphafold showing the C2 domain (green) and the catalytic domain (orange). (D) AND gate logical representation showing the conjunction of both inputs – Nuclear Ca^2+^ and INM tension as requirements for nuclear cPLA_2_ activation.

PLA_2_s like cPLA_2_, hydrolyze the sn-2 acyl chain of phospholipids to generate free fatty acids and lysophospholipids. To this end, PLA_2_ enzymes insert hydrophobic residues (located in their catalytic domain and/or dedicated membrane binding motifs) into the lipid bilayer (Figure 1) [15]. Hydrophobic insertion renders PLA_2_s, and perhaps many other peripheral proteins [16,17], effective membrane stress sensors. Tightly packed headgroups hinder their adsorption [6], whereas loosely packed bilayers display hydrophobic lipid-packing defects (LPDs) that serve as adsorption sites. LPDs may arise from positive curvature [18–20], conical lipids [21], and mechanical stretch (Figure 1). Owing to distinct lipid composition, LPDs are more abundant on endomembranes than on the PM [22]. However, on nuclear membranes that are relatively uncurved compared to nanometer-sized, vesicular organelles, membrane tension typically dominates LPD formation, especially on short timescales. Tension also thins bilayers, creating hydrophobic mismatch with transmembrane proteins and promoting conformational changes such as channel opening [1]. Hence, cPLA_2_, just like mechanosensitive channels, registers bilayer tension as ‘force-from-lipid’ via hydrophobic membrane interactions.

Besides LPDs, cPLA_2_ requires Ca^2+^ [23–30] for electrostatic bilayer engagement of its N-terminal C2 domain (Figure 1). Bilayer tension drastically lowers the Ca^2+^ threshold for its hydrophobic insertion [31]. cPLA_2_ prefers phosphatidylcholine [32], which is abundant in ER and nuclear membranes [33], and selectively releases arachidonic acid (AA), the metabolic precursor of the eicosanoid class of bioactive lipids, which comprise prostaglandins, leukotrienes and many potent G-protein coupled receptor ligands [34]. Downstream of AA release, the redox-sensitive eicosanoid 5-KETE [35] mediates rapid leukocyte recruitment to hypotonically shocked zebrafish wound margins and infection sites [10,36]. Multiple eicosanoid enzymes are constitutively nuclear, underscoring the biomedical importance of nuclear eicosanoid metabolism [37] and its regulation by nuclear deformation [10,11,31,38–42].

Research into the cPLA_2_ pathway points to the INM as a privileged mechanosensitive surface. Disrupting constitutive nucleoplasmic localization of zebrafish cPLA_2_ compromises confinement sensing [39], implying enrichment of at least one of its two activating cues, Ca^2+^ or tension/LPDs, in deformed nuclei. Because Ca^2+^ equilibrates rapidly through nuclear pores [43], INM tension is likely decisive. New data suggest that the Ca^2+^ -insensitive Amphipathic Lipid Packing Sensor (ALPS) probe accumulates on the INM when expressed in the nucleoplasm, but when cytoplasmic it rather targets curved vesicles and show little binding to the outer nuclear membrane during swelling [41]. Unlike the nucleoplasm, the cytoplasm contains a lot of highly curved, LPD-rich endomembranes in the absence of any physical stimulation. Those adsorption-prone membranes are excluded from the nucleus, which instead relies on deformation and INM tension to create *‘ad hoc’*-LPDs on shorter timescales. At least in principle, nuclear cPLA_2_ can thus distinguish chemical signals (e.g., GPCR or purinergic receptor agonists) that increase Ca^2+^ but not LPDs, from physical cell body stimulation that elevates both Ca^2+^ and LPDs. Such physicochemical AND-gating (Figure 1D) may allow cells to gather complex, ‘proprioceptive’ information from their physical environments [39].

If nucleoplasmic residency is essential for mechanosensitivity, how is cPLA_2_’s nuclear import regulated? Bioinformatics predictions indicate robust Nuclear Localization Sequence (NLS) motifs in zebrafish cPla_2_ [44], consistent with its predominant nucleoplasmic localization even when ectopically expressed in cultured mammalian cells [10,11,38]. Human cPLA_2_, with weaker predicted NLSs, is mostly cytoplasmic and enters nuclei context-dependently; the Human Protein Atlas reports nucleoplasmic cPLA_2_ in the urothelium, skin, and uterine smooth muscle [45], i.e., tissues at least occasionally exposed to mechanical stress. As nucleocytoplasmic transport is also modulated by the physicochemical properties of the cargo itself [46,47], it seems worthwhile testing whether potential differences in the mechanical stability of NLS-proximal protein regions, perhaps along with posttranslational modifications, may account for the species differences in baseline subcellular cPLA_2_ localization. The nuclear shuttling of many signal transducers, such as YAP, itself turns out to be mechanically gated (see below) [48,49], and nuclear cPLA_2_ increases in dendritic cells when confined [50]. It is yet to be determined if human cPLA_2_, which is larger than YAP, similarly shuttles across the nuclear pore in a mechanically controlled manner (via facilitated transport). The possibility of a second mechanical checkpoint upstream of INM adsorption is intriguing. Interestingly, both cPLA_2_ and Ca^2+^ -independent iPLA_2_ concentrate in nuclei during caspase-independent death after hypoxia [51]. Dying cells exhibit marked nuclear deformations with elevated INM tension [40,41]; but whether or how cell death/damage associated mechanical membrane stress governs cPLA_2_’s nuclear shuttling remains to be addressed. Nuclear cPLA_2_ also rises in subconfluent, proliferative cells and responds to phosphorylation and transport modulators [52,53]. Serum starvation reduced nuclear cPLA_2_ in EA.hy.926 endothelial cells but increased it in rat mesangial cells [54], highlighting context dependence. Finally, inactive N-terminal cPLA_2_ fragments may also accumulate in the nucleus after caspase-mediated cleavage [55–57], e.g., during apoptosis or the cell cycle, potentially behaving as competitive membrane-binding inhibitors of intact PLA_2_’s. Such proteolysis-dependent re-localization must be carefully distinguished from nucleocytoplasmic shuttling of active, full-length cPLA_2_.

Known physiological roles of the cPLA_2_-mediated mechanotransduction span cell differentiation, migration, and proliferation, along with auto-/paracrine immune signaling. In the musculoskeletal system, prostaglandin E_2_ is a classic mediator of load responses [58–81]. Historically, mechanosensitive prostaglandin synthesis has been mainly ascribed to stress-induced cyclooxygenase-2 transcription, and the regulation of the rate-limiting step, i.e., PLA_2_-mediated AA release, has been less considered. Recent data now implicates cPLA_2_ in mechanosensitive chondrogenesis from mesenchymal stem cells [82].

Turning to damage signaling, various studies implicate the cPLA_2_-NMMT pathway in pre- and post-lytic ‘alarmin’ function [83]. Hypotonic shock and ferroptosis trigger INM adsorption of cPLA_2_ and rapid AA release from stressed tissues, respectively [10,11,40,84–87]. Resulting eicosanoids recruit antimicrobial leukocytes and promote serum exudation, conferring acute tissue protection and restoration [10,11,14,36,42]. After lysis, intranuclear Ca^2+^ surges and extranuclear colloid osmotic pressure collapses, inflating nuclei and reinforcing cPLA_2_-INM adsorption [11,41]. Consistently, digitonin-permeabilized HeLa corpses placed at ‘isotonically silenced’ zebrafish wounds attract neutrophils in a manner dependent on nuclear cPLA_2_ and membrane tension [11,88].

In cell migration, cPLA_2_-NMMT aids mechanoadaptation. Cancer, immune, and embryonic cells migrate mesenchymally on planar substrates but switch to bleb-based amoeboid movement in confinement, a transition promoted by nuclear compression [89,90]. cPLA_2_-NMMT is one driver of this mesenchymal-to-amoeboid transition [38,39], along with other, NMMT-independent mechanisms [91]. Relatedly, confinement-induced cPLA_2_ accumulation inside the nuclei of dendritic cells promotes the expression of the chemotactic chemokine receptor CCR7 via an autocrine PGE_2_/NFκB loop, which may promote tolerogenic lymph-node homing [50]. It is at least intriguing to speculate that some of the gastrointestinal ulceration seen in cPLA_2_ deficiency [92] might be caused by lack of tolerogenic signaling.

Finally, cPLA_2_-NMMT intersects with cell-cycle control. Nuclear deformation regulates shuttling of key factors [49]. PLA_2_ inhibition or sequestration of cPLA_2_ at the Golgi has been linked to impaired endothelial proliferation [93], and a recent report suggests that cPLA_2_ knockdown or inhibition inhibits the proliferation of mechanically stretched keratinocytes [94]. During prophase, actomyosin-driven compression stretches the nuclear envelope, recruits cPLA_2_, and facilitates CDK1 import; blocking phospholipase activity prevents CDK1 entry and delays mitosis [95]. The detailed mechanism by which cPLA_2_ primes CDK1 nuclear access in flattened nuclei remains to be defined. Given the differential accumulation of cPLA_2_ on the NE or in the nucleo/cytoplasm depending on physiological context, it will be critical to establish the role of cPLA_2_-NMMT in generating fate decisions at the tissue level *in vivo*.

### The nuclear pore complex

Apart from the cPLA_2_ pathway, another NE component that has been widely reported to respond to membrane tension is the Nuclear Pore Complex (NPC). NPCs are massive macromolecular complexes of over 100 MDa in humans, that regulate transport between the nucleus and cytoplasm. They are formed by proteins called nucleoporins or nups, and have a cylindrical shape that crosses both nuclear membranes, supported by a structural scaffold [96,97]. This scaffold is composed of two ‘outer’ rings on the cytoplasmic and nucleoplasmic side, and an inner ring in between. From this ring structure a series of proteins with disordered domains emanates toward the central channel of the pore. These proteins, called FG nups due to the presence of phenylalanine and glycine (FG) repeats, form a meshwork that acts as a barrier to free diffusion (called the permeability barrier) and thereby control how transport occurs [98–100].

Conclusive evidence now demonstrates that NPCs can change their conformation, and that this occurs in response to mechanical signals. The NPC inner ring is formed by eight symmetric spokes [96,97] that are linked to each other through flexible linker proteins [101]. This flexibility allows NPCs to dilate and constrict by changing the distance between the spokes. This mechanism was proposed more than a decade ago [102] and has since been demonstrated experimentally. Indeed, changes in NPC diameter have been observed with transmission electron microscopy (EM) in fibroblasts in response to substrate stiffness [48], with Cryo EM in yeast in response to osmotic shocks [103], and with Cryo EM in cancer cells in response to ECM adhesion [104]. The variety of mechanical stimuli leading to NPC dilation strongly suggests that the key parameter is tension in the NE. However, an important unresolved question is how force is transmitted from the NE to NPCs. This could involve some of the NPC components with described links to either the nuclear lamina or the Linker of Nucleoskeleton and Cytoskeleton (LINC) complex, such as nup153 [105,106], nup53 [107], TPR [108], or POM121 [109,110]. Recently, a molecular force sensor based on Förster resonance energy transfer (FRET) showed that the NPC component nup210 is subjected to force and is sensitive to different mechanical perturbations [111]. Interestingly, the results suggest that the key mechanical parameter regulating force transmission is nuclear strain (which should directly correlate to NE tension), rather than a specific cytoskeletal component.

As one would expect intuitively, changes in the conformation of NPCs affect molecular transport through them. Passive diffusion through NPCs (which occurs symmetrically in both directions) increases or decreases when NPCs dilate or constrict in response to hypo- or hyper-osmotic shocks, respectively (Figure 1) [48,103,112]. Osmotic shocks also affect pressure-driven hydrodynamic flows through NPCs [113]. Passive diffusion also decreases when actomyosin tension in cells is reduced, either because they are seeded on soft rather than stiff substrates, or because the mechanical link between the actomyosin cytoskeleton and the nucleus is abrogated by interfering with the Linker of Nucleoplasm and Cytoskeleton (LINC) complex [112].

NPC conformation affects not only passive diffusion but also active transport through NPCs. Passive diffusion decreases with molecular weight and becomes very slow for molecules above ~40 KDa [114]. Transport of larger molecules requires active nucleocytoplasmic transport, which is mediated by complex molecular machinery, reviewed in detail elsewhere [115]. Summarizing, cargo molecules undergoing active transport possess specific peptide sequences called either Nuclear Localization Sequence (NLS) or Nuclear Export Sequence (NES), which mediate nuclear import and export, respectively. NLS and NES sequences bind, respectively, to importin or exportin proteins. Due to specific interactions with FG-nups, importins and exportins can diffuse through NPCs even if they carry large cargoes. The binding and unbinding between cargo molecules and importins/exportins depend on the small GTPase RAN, which can in turn bind to either GTP or GDP. This GTP/GDP binding status exhibits a steep gradient across the NE, with RAN-GTP dominating in the nucleus and RAN-GDP in the cytoplasm. In this manner, transport directionality is enabled by the fact that RAN-GTP impairs cargo-importin binding but favors cargo-exportin binding. Importantly, this means that the active, energy consuming step in active transport is the maintenance of this gradient, whereas actual transport through NPCs remains diffusive and non-directional. For this reason, it is referred to as ‘facilitated diffusion.’

Since active transport is in fact also diffusive, it is intuitive to expect that it will also be affected by NPC dilation. Indeed, the rates of facilitated diffusion both in the import and export direction increase with mechanical force application to the nucleus, either done directly by deforming nuclei with Atomic Force Microscopy (AFM), or in response to changes in substrate stiffness or LINC Complex engagement [112]. Thus, NPC dilation affects both passive and facilitated diffusion, but in different ways. For passive diffusion, increased molecular weights strongly decrease both diffusion rates and their mechanical sensitivity, with high molecular weight (MW) cargoes exhibiting very low diffusivities that are no longer affected by NPC dilation. In contrast, facilitated diffusion has a much weaker dependency on MW, with large cargoes retaining both diffusion and sensitivity to NPC dilation. In this manner, cargo molecules with properties enabling both passive diffusion (because of their MW) and facilitated diffusion (because of NLS/NES sequences) will respond differently to both types of transport. This generates an asymmetry which implies that force applied to the NE can lead to molecular translocation either to the nucleus (for cargoes with NLS sequences) or to the cytoplasm (For cargoes with NES sequences) [112]. The mechanistic details by which NPC dilations affects the conformation and permeability properties of FG-nups have been analyzed in silico [116,117] but remain to be studied in detail experimentally. In this regard, it is important to note that diffusive properties through NPCs do not only depend on MW and the presence of NLS/NES sequences, but also on the presentation of specific residues on protein surfaces [118] and on protein mechanical stability [46,47]. Furthermore, the properties of molecules involved in the transport machinery (such as importins) could also be mechanosensitive on their own [119].

The mechanical regulation of transport has important implications in cell function. For instance, the mechanism of force-mediated nuclear molecular translocation described above can explain, at least in part, the mechanosensitivity of major transcriptional regulators, such as YAP [48], Twist1, snail, and SMAD3 [112]. These transcriptional regulators are involved in important pathophysiological processes in cancer, development, and other scenarios, and they all respond to tissue stiffness [120–123]. Beyond the well-studied mechanical paradigm of tissue stiffness, the fact that the mechanosensitive mechanism is mediated by the NE means that molecules such as YAP also respond to other mechanical stimuli involving nuclear deformations, such as tissue curvature [124] and nuclear compression [125]. Interestingly, the recent development of mechanosensitive probes based only on nuclear transport [125] now allows to dissect how the mechanosensitivity of transcriptional regulators depends on nucleocytoplasmic transport, versus other mechanisms such as binding to cytoskeletal components as in MRTF-A [126], or force-dependent binding to chromatin [127,128].

Apart from transcriptional regulation, changes in nuclear transport mediated by mechanics also regulate nuclear volume [129]. There is also an intriguing effect of the neurodegeneration-related protein tau, which binds to NPCs through nup98 [130,131], in generating nuclear envelope invaginations that lead to mRNA accumulation [131]. Beyond these examples and given the central role of nucleocytoplasmic transport in cell biology, the implications of its mechanical regulation could be vast. A particularly intriguing process that remains to be studied in this context is RNA export.

### Nuclear envelope tension

Because cPLA_2_- and NPC-mediated nuclear mechanotransduction are regulated by nuclear-envelope (NE) tension, it is essential to understand how NE tension is controlled and how it can be measured.

**Figure 2.**
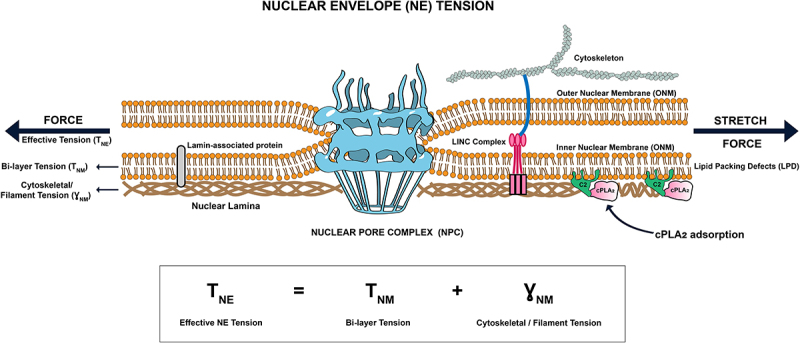
Force integration in the nuclear envelope. Schematic shows how mechanotransduction via cPLA_2_ and NPCs requires the integration of both force-from-lipid (bi-layer tension) and force-from-filament (cytoskeletal/filament tension) to sense the effective tension in the nuclear envelope (NE). How the nuclear lamina is linked to both the INM and NPCs remains to be fully elucidated.

The NE is a composite structure of two lipid bilayers – the outer and inner nuclear membranes (ONM/INM) – fused at nuclear pores and mechanically stabilized by an intermediate-filament lamina composed of lamins A/C and B1/B2 (Figure 2) [3]. Forces applied to the nucleus first flatten preexisting NE undulations, then deform the elastic lamina, and finally stretch the lipid bilayers. NE stretch extends/dilates NPCs [103,113,132], and generates lipid-packing defects (LPDs) that promote peripheral membrane protein adsorption. Mechanochemical pathways driven by direct bilayer remodeling have been termed ‘force-from-lipid.’ When force alters membrane-associated cytoskeletal elements (e.g., by exposing interaction sites in the lamina) [1], the pathway is ‘force-from-filament.’ A third mode – ‘force-from-chromatin’ – might be admittable if nuclear deformation directly, i.e., without intermittent signaling, modulates gene transcription [127].

For nuclear mechanotransduction via cPLA_2_ and NPCs (see above), as for ion-channel or lysis-pore mechanotransduction at the plasma membrane (PM) [133,134], both ‘force-from-lipid’ and ‘force-from-filament’ paradigms apply. Disentangling their contributions is challenging and may be an inadequate abstraction in some cases. Like in the PM, NE lipids are mechanically coupled to cytoskeletal filaments (e.g., through lamin B1, LINC complex, etc.). As composite material, the NE may thus show emergent properties not found with its isolated components. By analogy to the PM [135], the ‘apparent’ or ‘effective’ NE tension (T_NE_, N/m) can be described as force required to stretch this composite and mathematically expressed by the sum of in-plane bilayer tension, (T_NM_, N/m), and cytoskeletal/filament tension ($\bar{x}$_NM_, N/m) (Figure 2). The individual contributions of T_NM_ and $\bar{x}$_NM_ to mechanotransduction are subjects of intense research and discussion. The above used term ‘NMMT’ refers to nuclear membrane mechanotransduction primarily controlled by T_NM_ as opposed to $\bar{x}$_NM_—but again, both types of tensions are inherently interdependent.

An interesting open question concerns the length and time scales of NE tension propagation along the nucleus. At the plasma membrane, tension can propagate within seconds across the entire cell surface if the underlying actin cortex is also mechanically engaged [136]. However, tension applied only to the lipid membrane does not propagate as it is dampened by the cortex [137]. The very different nature of the NE (double versus single lipid bilayer, different lipid composition, and an underlying cytoskeleton made of passive intermediate filaments rather than an active actin cortex) may lead to a very different behavior in response to mechanical stress. For example, nuclear blebbing due to local defects in the nuclear lamina and aberrant chromatin organization is a result of heterogenous tension distribution across the NE [138–140]. Consequently, the effects of various cellular stressors on NE tension remain to be explored. To study this experimentally, T_NE_ can be probed with atomic force microscopy, tether pulling using optically trapped beads, or micropipette aspiration [135]. In nuclear mechanobiology, a key caveat is that isolating nuclei from cells (e.g., by cytoskeletal softening and centrifugation) will surely alter their membrane and biomechanical properties, e.g., by removing portions of their endoplasmic reticulum (ER) membrane reservoir. Micro-harpooning enables direct intracellular probing of T_NE_ but still involves PM puncture [141]. Noninvasive readouts of bilayer tension T_NM_ employ fluorescent probes. Generalized-polarization (GP) dyes such as C-Laurdan and Di-4-ANEPPDHQ incorporate near the water – lipid interface and shift emission with membrane hydration, which increases with looser lipid packing (i.e., with in-plane tension) [142]. Amphipathic helices also sense lipid packing in the inner nuclear membrane [41,143]. Organelle-targeted Laurdan analogues, in principle, allow compartment-specific measurements [144]. Flipper-TR dyes (also available as organelle-targeted versions) insert deeper into the bilayer core as GP dyes, and report on tension-dependent changes in lipid packing and phase behavior via intramolecular (de)planarization that can be quantified by fluorescence-lifetime imaging [145]. Finally, membrane adsorption of genetically encoded LPD sensors such as cPLA_2_-mKate2 or ALPIN (Amphipathic Lipid Packing sensor Inside the Nucleus) provides a proxy-readout for INM tension (T_INM_) in intact cells [11,31,38,39,41,42]. For all these probes, an important limitation is that they sense T_NE_ indirectly (for instance via lipid packing) and therefore can potentially respond to non-mechanical factors. This list of current measurement options will likely grow larger in the future, probably also improving in specificity. T_INM_ increases when pressure-driven nuclear-volume changes (e.g., during osmotic swelling or compression) outpace the acquisition of new surface area. Additional area may be supplied by flattening thermal undulations and larger stabilized folds, or by compensatory lipid flow from the ER. However, such flow must navigate membrane-anchored ‘obstacles’ or ‘valves’ [137,146] and potential topological bottlenecks such as nuclear pores. Beyond the ER, NPCs themselves may constitute a structurally stabilized membrane reservoir that buffers or dissipates excess NE tension [147] — a speculation recently supported by compelling structural evidence [132]. Whereas large folds and undulations can prevent NE tension from arising during volume stress, the ER and NPCs act as ‘feedback reservoirs’ that are engaged only once tension builds, in analogy to similar phenomena at the plasma membrane [148]. The availability of these reservoirs may be further regulated by factors beyond NE tension, including NPC material properties (e.g., elasticity, stability) [132] and ER topology and contiguity with the nucleus. For example, during stress, Ca^2+^ can transiently or permanently disrupt NE – ER continuity, elevating T_INM_ [41]. This stress-dependent loss of nuclear-membrane reservoirs may convert heavily stressed, dying, or dead cells into bioactive-lipid signaling hubs via cPLA_2_-dependent arachidonic-acid release [11,41].

## Outlook

Here, we have reviewed the two most studied paradigms of mechanotransduction mediated directly by the NE lipid bilayer: the cPLA_2_ pathway and NPCs. If we take guidance from the much better studied plasma membrane, with a vast diversity of characterized mechanotransduction mechanisms [4], it is likely that these two examples are only the tip of the iceberg. Indeed, recent research suggests that amphipathic helices could also act as NE mechanosensors [41,143], and further mechanisms are likely to be revealed in upcoming research. In this regard, it will be crucial to understand the mechanical properties of the NE, which will clarify which types of mechanical stimuli the NE is suited to sense, and at what length and time scales.

## Data Availability

No data was generated in this work.
